# Inhaled Lactonase Reduces *Pseudomonas aeruginosa* Quorum Sensing and Mortality in Rat Pneumonia

**DOI:** 10.1371/journal.pone.0107125

**Published:** 2014-10-28

**Authors:** Sami Hraiech, Julien Hiblot, John Lafleur, Hubert Lepidi, Laurent Papazian, Jean-Marc Rolain, Didier Raoult, Mikael Elias, Mark W. Silby, Janek Bzdrenga, Fabienne Bregeon, Eric Chabriere

**Affiliations:** 1 Unité de Recherche sur les Maladies Infectieuses et Tropicales Emergentes, UMR CNRS-IRD 6236, IFR48, Aix-Marseille Université, Marseille, France; 2 Réanimation - Détresses Respiratoires et Infections Sévères, APHM, CHU Nord, Marseille, France; 3 Department of Biology, University of Massachusetts Dartmouth, Dartmouth, Massachusetts, United States of America; 4 Department of Emergency Medicine, Alpert School of Medicine, Providence, Rhode Island, United States of America; 5 Weizmann Institute of Science, Biological Chemistry, Rehovot, Israel; Ben-Gurion University of the Negev, Israel

## Abstract

**Rationale:**

The effectiveness of antibiotic molecules in treating *Pseudomonas aeruginosa* pneumonia is reduced as a result of the dissemination of bacterial resistance. The existence of bacterial communication systems, such as *quorum* sensing, has provided new opportunities of treatment. Lactonases efficiently quench acyl-homoserine lactone-based bacterial *quorum* sensing, implicating these enzymes as potential new anti-*Pseudomonas* drugs that might be evaluated in pneumonia.

**Objectives:**

The aim of the present study was to evaluate the ability of a lactonase called *Sso*Pox-I to reduce the mortality of a rat *P. aeruginosa* pneumonia.

**Methods:**

To assess *Sso*Pox-I-mediated quorum quenching, we first measured the activity of the virulence gene *lasB*, the synthesis of pyocianin, the proteolytic activity of a bacterial suspension and the formation of biofilm of a PAO1 strain grown in the presence of lactonase. In an acute lethal model of *P. aeruginosa* pneumonia in rats, we evaluated the effects of an early or deferred intra-tracheal treatment with *Sso*Pox-I on the mortality, lung bacterial count and lung damage.

**Measurements and Primary Results:**

*Sso*Pox-I decreased PAO1 *lasB* virulence gene activity, pyocianin synthesis, proteolytic activity and biofilm formation. The early use of *Sso*Pox-I reduced the mortality of rats with acute pneumonia from 75% to 20%. Histological lung damage was significantly reduced but the lung bacterial count was not modified by the treatment. A delayed treatment was associated with a non-significant reduction of mortality.

**Conclusion:**

These results demonstrate the protective effects of lactonase *Sso*Pox-I in *P. aeruginosa* pneumonia and open the way for a future therapeutic use.

## Introduction

The dramatic increase of antibiotic resistance in bacterial isolates from intensive care unit (ICU) patients leads to an important reduction of therapeutic arsenal [1] [2], [3] [4], [5]. Alternative approaches to combat multidrug-resistant strains are under extensive research, especially using molecules that can reduce bacterial expression of virulence factors [1]–[3] [6].

Expression of virulent phenotypes of numerous pathogenic bacteria is activated when the pathogen senses that a critical population density has been reached. This ability relates to the *quorum* sensing (QS) by which bacteria can sense messenger molecules thus virulence genes are activated [7]. This communication ability enables the bacteria to coordinate bacterial population behavior during the invasion of a host [8] [9].

Acyl-Homoserine Lactones (AHLs) are QS molecule messengers used by a large number of Gram-negative bacteria, including *Pseudomonas aeruginosa* [10]. These bacteria are able to form biofilms whose maturation is regulated through QS [11]. Biofilms have been implicated in antibiotic resistance in chronic *P. aeruginosa* infections, primarily through the limitation of the diffusion of antibiotics into the bacterial colonies [12]. *P. aeruginosa* possesses two different AHL-based QS systems: the LasI/R and RhlI/R with their respective cognate 3-oxo-C12 AHLs and C4 AHLs messenger molecules [13].

The inhibition of bacterial virulence by targeting QS mechanism can be mediated by (i) the inhibition of the messenger production, (ii) inhibition of its detection, both known as QS inhibition/inhibitors [14], [15] [16], or (iii) the elimination of the messenger known as *quorum* quencher (QQ) [17], [18] thus alteration of the *P. aeruginosa* QS system can attenuate its virulence. In pneumonia, the efficacy of QS modulation has been tested at the genomic level with reduction in lethality of infected animals [9]. Therapeutic management of animals with pneumonia has been tested with QS inhibitors such as furanones and patulin showing that these molecules can increase the clearance of bacteria in mice infected with *P. aeruginosa* [19], [20] [21]. Because some *in vitro* studies suggest toxicity of furanones and patulin [22] [23], other QS inhibition options are under investigation [24] among which the use of lactonases or acylases enzymes [10] [25]. Lactonases efficiently decrease the production of virulence factors and biofilm by *P. aeruginosa in vitro* [26] [27], and may therefore represent an alternative and promising strategy for reducing bacterial virulence. The efficacy of lactonases to decrease bacterial virulence has been reported in several hosts [28], [29] [30]. In a recent *in vivo* study on mice with pneumonia [31], the use of a lactonase-producing *P. aeruginosa* mutant showed reduced lung injury and increased survival as compared to the wild strain. These encouraging results would suggest that the use of lactonases as a topic therapeutic agent could be effective in improving outcome in *P. aeruginosa* pneumonia.

The aim of our work was to test the efficacy of inhaled lactonase in improving survival of rats with acute *P. aeruginosa* pneumonia. To do this, we used an engineered variant of the hyperthermostable lactonase *Sso*Pox (first isolated from the extremophilic archaea *Sulfolobus solfataricus*) [32], *Sso*Pox-I, exhibiting a high stability and an improved ability to hydrolyze 3-oxo-C12 AHLs. We first tested *in vitro* the ability of our molecule to reduce, in a *P.aeruginosa* PAO1 strain, the virulence gene *lasB* activity, the pyocianin synthesis, the proteolytic activity and the biofilm formation. Thus, we evaluated the effect of *Sso*Pox-I when delivered early or late intra-tracheally to animals with lungs infected with *P. aeruginosa.*

## Material and Methods

### Protein production & purification

The plasmid encoding *Sso*Pox-I protein was commercially obtained (GeneArt, Invitrogen; Germany). *Sso*Pox-I protein was synthesized in *E. coli* strain BL21(DE_3_)-pGro7/GroEL (TaKaRa) in ZYP medium [33] containing 100 µg/ml ampicillin and 34 µg/ml chloramphenicol as previously described [22]. The proteins were purified as previously described [34]. Briefly, the culture was incubated at 70°C for 30 minutes, followed by differential ammonium sulfate precipitation, dialysis and exclusion size chromatography. The proteins were quantified using a nanospectrophotometer (Nanodrop, ThermoFisher Scientific, France) and the protein molar extinction coefficient was determined using the protein primary sequence in PROT-PARAM (ExPASy tool software) [35]. The protein yield was approximately 10 mg/L for *Sso*Pox-I, and the identity and purity of the purified protein was assessed through sodium dodecyl sulfate polyacrylamide gel electrophoresis (SDS-PAGE) and mass spectrometry (Plateforme Timone, Marseille, France). The enzymes were stored and used in phosphate-buffered saline (PBS) (Biomerieux; France).

### *In vitro* experiments

#### P. aeruginosa culture

*P. aeruginosa* (strain PA01 ATCC 15692) was grown at 37°C in Luria-Bertani (LB) medium (BD, France) with shaking (200 rpm). When required, 1.5% bacto agar was added to solidify the LB.

For *in vivo* experiments [36], aliquots containing *P. aeruginosa* PAO1 strain were thawed and cultured on COS (Biomerieux, France) (Columbia with 5% Sheep blood) agar plates. Ten fresh PAO1 colonies were sampled and cultured at 37°C in tryptic soy broth (TSB, Biomerieux, France) with continuous shaking until the OD_600 nm_ = 1. Serial dilutions were subsequently performed to adjust the bacterial amount, and the precise concentrations were confirmed after plating serial dilutions on the appropriate culture medium and counting the resulting colonies.

#### LasB reporter system

A 50-µl aliquot from an 18-hour culture of *P. aeruginosa* PAO1 carrying P*lasB*-*luxCDABE* (QS reporter) was added to the wells of a 96-well plate. A ten-fold dilution series from 50 µg to 0.05 µg of *Sso*Pox-I was added to the wells containing the *P. aeruginosa* reporter strain. LB was added to each well to generate a final volume of 100 µl. The plates were incubated at 37°C for 90 minutes, with shaking every 10 minutes, and subsequently analyzed using a Varioskan Flash multimode plate reader. The luminescence was measured every 10 minutes to determine the QS reporter activity.

#### Quantification of pyocianin synthesis

Quantification of pyocyanin was performed as previously described [37]. Briefly, *P. aeruginosa* PAO1 strain was grown in 1 mL of Glycerol-alanine (GA) minimum medium (10 mL.L^−1^ glycerol, 6 g.L^−1^ L-alanine, 2 g.L^−1^ MgSO_4_, 0.1 g.L^−1^ K_2_HPO_4_, 0.018 g.L^−1^ FeSO_4_) with and without 14 µM of filtered (0.2 µm pore) *Sso*Pox-I enzyme during 24 h at 37°C and 450 rpm shaking in 48-well plate (Greiner Bio-One, Germany). Cells were centrifuged 15 min at 12.000 *g* and the supernatant was filtered (0.2 µm pore). Extraction of pyocyanin was performed on 500 µL of supernatant using 0.5× volume of chloroform and absorbance was subsequently measured at 690 nm. Blank assay was realized using 500 µL of culture medium with and without 14 µM of filtered (0.2 µm pore) *Sso*Pox-I enzyme.

#### Proteolytic activity

Measurement of the proteolytic activity was made using azocasein enzymatic assay as previously described [38]. Briefly, *P. aeruginosa* PAO1 strain was grown in 200 µL of LB medium with and without 14 µM of filtered (0.2 µm) *Sso*Pox-I enzyme during 24 h at 37°C and 450 rpm shaking in 96-well plate. The *Sso*Pox-I enzyme stock solution being in PBS buffer, control cultures were supplemented with equivalent PBS quantity. Cells were centrifuged 15 min at 12.000 *g*. The reaction was performed in 0.3 M TrisHCl buffer (pH 7.5) with 50 µL of azocasein (Sigma, St. Louis, USA) (30 mg.mL^−1^ dissolved in water) and with 50 µL of culture's supernatant for a final volume of 1.5 mL. The reaction was incubated at 37°C for 1 h and subsequently stopped by addition of 250 µL of 20% (w/v) trichloroacetic acid (TCA). The blank assay was realized using 50 µL of culture medium with and without 14 µM of filtered (0.2 µm pore) *Sso*Pox-I enzyme. After centrifugation at 12.000 *g* for 10 min, optical density was measured at 366 nm. The proteolytic activity was defined as the increase in absorbance at 366 nm.h^−1^ per number of cells (OD_600_).

#### Biofilm formation assays

Liquid cultures of *P. aeruginosa* PAO1 were grown for 18 hours, and subsequently diluted 1∶50 in 10% TSB. To examine biofilm susceptibility to *Sso*Pox-I, 100 µl aliquots were dispensed onto Calgary Biofilm Device 96-well plates (MBEC Assay for Physiology & Genetics, Innovotech Inc., Edmonton, Alberta, Canada). A three-fold dilution series from 50 µg to 0.5 µg of *Sso*Pox-I was added to the wells containing *P. aeruginosa*. The plates were incubated for 4 hours with rocking at 120 Hz at 37°C, and subsequently, the MBEC device with adherent *P. aeruginosa* biofilms was placed on a fresh 96-well plate containing 100 µl of 1% crystal violet dye in each well for 15 minutes. The MBEC device was subsequently washed three times with 100 µl of water to remove excess dye and allowed to dry. Crystal violet stain was solubilized from the biofilms after placing the MBEC device on a 96-well plate with 100 µl of 100% ethanol in each well. The solubilized crystal violet dye was measured at 600 nm using a Varioskan Flash multimode plate reader (Thermo). The optical density at 600 nm was also used to assess *P. aeruginosa* PAO1 planktonic growth on the original 96-well plate to determine the effect of *Sso*Pox-I on *P. aeruginosa* planktonic growth.

### *In vivo* experiments

#### Ethics statement

The experiments and protocols were performed in accordance with the European law and the French version of this law details the statutory requirements for the live animal experiments (articles R214-87 to R215-10 of Code Rural, law #76–629 from July 10^th^, 1976/law #2001-464 from May 29^th^, 2001 (published in JORF on May 31^st^, 2001)). Consistent with these laws, the experiments were performed under the direct control of the researcher authorized through the Préfecture-des-Bouches-du-Rhone Administration (authorization number: 13–437). The animal experiments were performed in accordance with ‘Animal Research: Reporting In Vivo Experiments’ (ARRIVE Guidelines http://www.nc3rs.org.uk) and the guidelines of the Guide for the Care and Use of Laboratory Animals. All animal experiments were authorized through the National Animal Ethics Committee («Comité National de Réflexion Ethique sur l′Expérimentation Animale (Comité d′éthique de Marseille)»). The experiments were performed in the Faculté de Pharmacie-Aix-Marseille University.

The animals were euthanized with an intra-peritoneal injection of a lethal dose of thiopental (Panpharma, France).

#### General procedures

Adult Sprague-Dawley male pathogen-free rats, weighing 250 to 300 g, were obtained from SAS Janvier (Le-Genest-St-Isle, France) and housed in individual plastic cages (4 animals per cage) in a ventilated pressurized cabinet (A-BOX 160, Noroit, Rezé, France) with free access to water and standard diet food. The rats were anesthetized with 5% Sévoflurane (Abbott, Rungis, France) in 100% oxygen (anesthetizing box, Harvard Apparatus, Les Ulis, France). The trachea was exposed, and they were intubated using a 16-gauge catheter for drug and/or bacterial administration. The awakened rats were housed under the same conditions and weighed daily. At the end of each experiment, the rats were euthanized with an intra-peritoneal injection of a lethal dose of thiopental (Panpharma, France).

#### Rat tolerance of inhaled *Sso*Pox-I

The tolerance to intra-tracheal treatment with *Sso*Pox-I was examined in a preliminary study on 3 groups of animals (n = 3 per group) receiving 250 µl of *Sso*Pox-I at a concentration of 0.1, 1 or 10 mg/ml and compared with 5 control animals receiving 250 µl of PBS. After the treatment, signs of bad tolerance of the molecule were investigated *i.e.* shortness of breathing, prostration and atony or weight loss above 10% from baseline. Spontaneous mortality was also recorded. One animal from each group was sacrificed after 6, 24 and 48 hours. The remaining animals were sacrificed after 48 hours. Subsequently, the lungs were removed, macroscopically examined, and preserved in formaldehyde for histological assessment of lung damage.

#### Rat respiratory infection model and *Sso*Pox-I treatment

Three groups of 20 animals were infected through intra-tracheal inoculation with 250 µl of a PBS solution containing 10^8^ CFU/ml of *P. aeruginosa* PAO1.

Among the 3 groups of infected rats, one group received immediately after infection 250 µl of PBS (non-treated group: NT), while another group was treated with 250 µl of *Sso*Pox-I at a concentration of 1 mg/ml (immediate treatment group: IT). The last group received 250 µl of 1 mg/ml *Sso*Pox-I at 3 hours after infection (deferred treatment group: DT). *Sso*Pox-I and additional PBS were delivered intra-tracheally using the same anesthetic procedure as used for the infection.

#### Lung processing and blood or spleen samples

After infection, the animals were observed for 2 days, and spontaneous mortality was examined. Animals' conditions and clinical status were checked every 2 hours. Humane endpoints were used during the survival study. If animals had one of the following signs, they were anesthetized and euthanized with an intra-peritoneal injection of a lethal dose of thiopental (Panpharma, France) to avoid suffering:

Major dyspnea with noisy breathing and head or neck movements associated with breathing
Prostration and atony
Weight loss>20% of initial body weight.

The remaining rats were euthanized after 48 hours. Subsequently, the lungs were removed aseptically. The right lung was homogenized in PBS for bacterial culture, and the left lung was preserved for histological analysis. The blood and spleen were sampled and cultured on agar plates to assess systemic diffusion of the bacteria.

#### Histological severity score (HSS)

Sections (3 mm thick) were obtained from the upper, mid and lower parts of the lungs, including the entire circumference. The sections were stained with hematoxylin and eosin. A pathologist blinded to the group identity (H. L.) examined the samples. The HSS was calculated based on the number of bronchopneumonia lesions (0, no lesions; 1, 30 lesions/lung; 2, ≥30 lesions/lung; 3, confluent lesions of bronchopneumonia), as previously reported [39], [40].

### Statistics

The number of studied animals (20 animals per group) was calculated based on a mortality reduction from 80% in the NT group infected with PAO1 to an expected mortality rate of 50% in the treated groups, with 90% statistical power and a two-sided alpha value of 0.05. The data were expressed as the means ± standard deviation (SD) or median [inter-quartile range, IQR] according to the distribution of the data. Student's *t*-test or the Mann-Whitney rank-sum test were used for inter-group comparisons. Kaplan-Meier analysis was performed to evaluate 48-h mortality. Intergroup differences were evaluated using the log rank test. The data analysis was performed with SPSS for Windows (Chicago, IL), version 12.0. A value of *p*≤0.05 was considered statistically significant.

## Results

### Protein production & purification

See Fig. S1 in supplementary material.

### *In vitro* experiments

**Figure 1:**
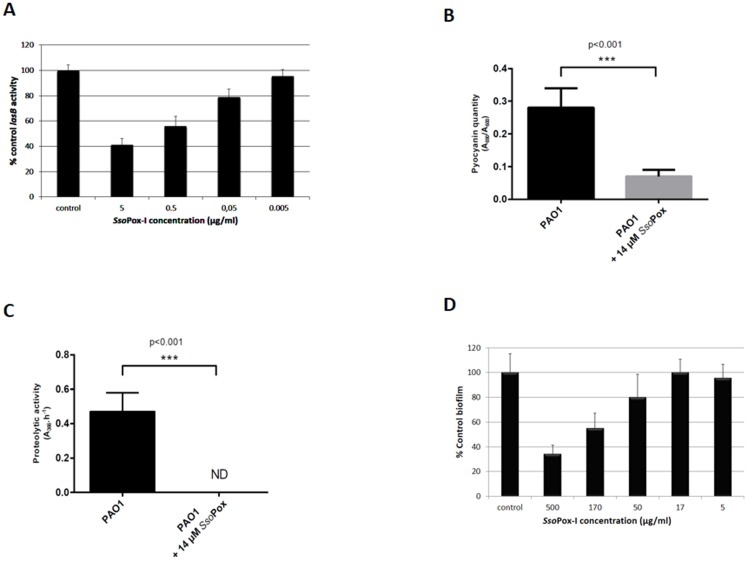
Effects of *Sso*Pox-I on PAO1 virulence factors expression and biofilm formation. 1A: *lasB* activity. The chart shows the *lasB* activity in treated cultures, expressed as the percentage of that in untreated controls (no *Sso*Pox-I), and these data represent the average of three independent experiments, each with three technical replicates. The error bars represent 95% confidence intervals. Student's t test p<0.05 for *Sso*Pox-I. T tests were used for the comparison of baseline with the highest dose of enzyme. **1B: pyocianin synthesis.** Pyocyanin quantification of *P. aeruginosa* PAO1 strain after 24 h growth in GA medium was assessed in presence (black) or absence (gray) of 14 µM of *Sso*Pox-I lactonase. Pyocyanin quantity was followed at A_690_ as per number of cells (A_600_). **1C: proteolytic activity.** Proteolytic activity of *P. aeruginosa* PAO1 strain after 24 h growth in LB medium was assessed in presence (black) or absence (grey) of 14 µM of *Sso*Pox-I lactonase. Proteolytic activity was measured by the azocasein assay (A_366_.h^−1^) as per number of cells (A_600_). ND stands for non-detected activity. **1D: biofilm formation.** Biofilms were grown in an MBEC device as described in the methods section. The dose-dependent inhibition of *P. aeruginosa* biofilm formation through *Sso*Pox-I was observed and analyzed using Student's t test p = 0.05 for *Sso*Pox-I.

We monitored the *lasB* activity in a *P. aeruginosa* PAO1 strain carrying the P*lasB*-*luxCDABE* plasmid. We showed that the addition of *Sso*Pox-I significantly reduced the levels of *lasB* activity (**Fig. 1A**). Moreover, this inhibition exhibited a dose-dependent profile with a half inhibition concentration ([C_1/2_]) of the enzyme of approximately 0.5 µg/ml (**Fig. 1A**).

Pyocyanin secretion was affected by the presence of *Sso*Pox-I and presented a 4 fold drop in quantity as compared to the control **(** **Fig. 1B** **)**.

Cultures of PAO1 made in presence of 14 µM *Sso*Pox-I showed after 24 h a clear drop of protease activity followed using azocasein assay. Protease activity in presence of *Sso*Pox-I was beyond detection limit **(** **Fig. 1C** **)**.

The biofilm assay showed that *Sso*Pox-I reduced biofilm formation in a dose-dependent manner with a [C_1/2_]∼170 µg/ml (**Fig. 1D**).

Interestingly, *Sso*Pox-I did not significantly affect the rate of *P. aeruginosa* growth. Indeed, even at the highest enzyme dose (5 mg/ml), the optical density of the cell culture did not significantly differ from that of the control experiment with no treatment (**Fig. S2**).

### *In vivo* experiments

#### Rat tolerance to inhaled *Sso*Pox-I

The effects of *Sso*Pox-I treatment on rat tissues were investigated. On the 9 rats that received *Sso*Pox-I into the trachea, none exhibited any sign of bad tolerance including in the group that received the highest dose. The weight curve of treated rats was not different to control animals and no animal loosed weight. There was no spontaneous mortality after 48-hours observation in the treated group as well as in the control group. After sacrifice, the lungs were harvested and the macroscopic examination showed no signs of injury. Histological assessment showed that there was no sign of lung damage 6, 24 or 48 hours after the treatment including in the group receiving the highest dose of *Sso*Pox-I.

#### Rat respiratory infection model and *Sso*Pox-I treatment

**Figure 2:**
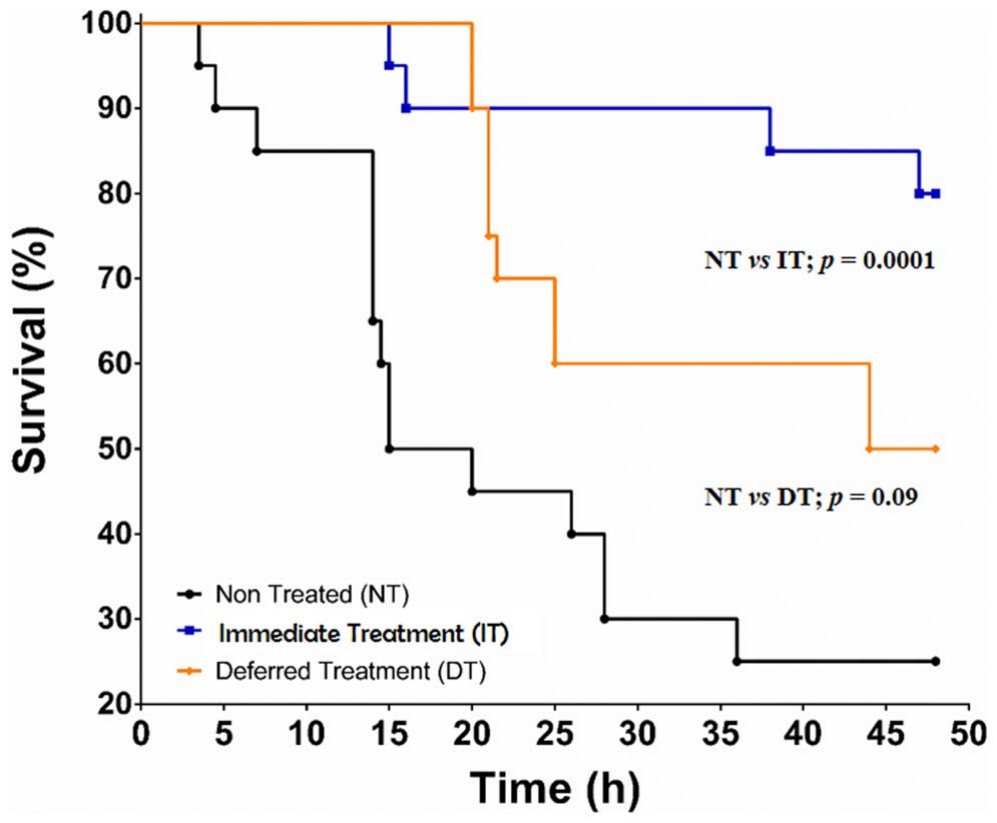
Forty-eight-hour survival curves for the 3 groups of animals after infection. The animals were infected with 10^8^ CFU/ml (2.5×10^7^ CFU/rat) of *P. aeruginosa* PAO1 in the following treatment groups: non-treated (NT), immediate-treatment (IT) or deferred treatment (DT) who received treatment with *Sso*Pox-I at 3 hours after infection.

The influence of *Sso*Pox-I on pulmonary *P. aeruginosa* infection was monitored in 3 groups of 20 rats. The spontaneous mortality rate was 75% (15/20) in the non-treated group (NT). When the rats were treated with *Sso*Pox-I (1 mg/ml) immediately after infection (IT), the mortality rate was significantly reduced to 20% (4/20) (*p* = 0.0001 vs. NT). The protective effect of lactonase on mortality was less significant in the deferred treatment (DT) group, where the treatment was administered at 3 hours after the infection (mortality rate of 50% (10/20) (*p* = ns vs. NT) (**Fig. 2**). However, in the DT group, the mean delay of mortality was significantly longer than that in the control group (respectively 26±9.5 vs. 17±9.2 hours; p = 0.04).

In addition to death, other parameters were monitored during the infection, including the weight of the animals. We observed that the loss of body weight, measured from the day of infection until the day of death, was significantly less important in the IT group than in the NT group (11.3±12 g vs. 20.4±9.3 g respectively; *p* = 0.01). The DT group lost 25.6±1.82 g of body weight (p = ns vs. NT group).

**Figure 3:**
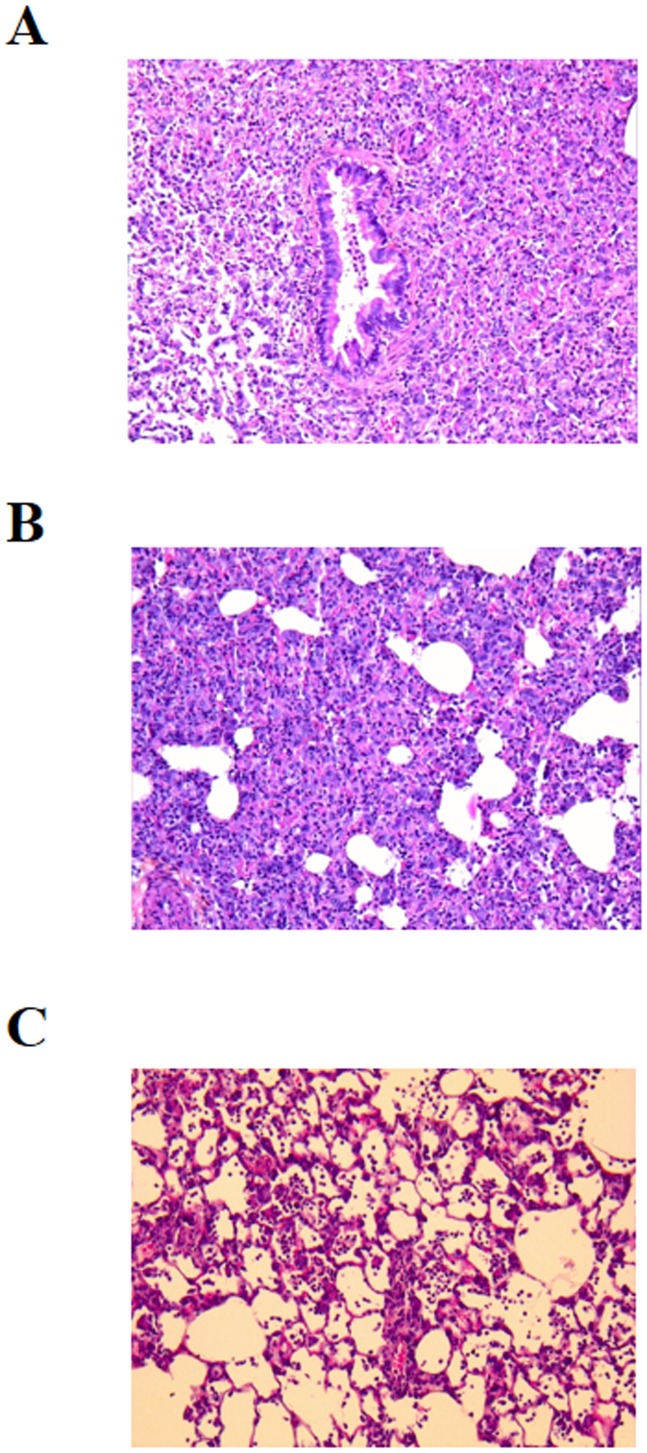
Lung histological examination after infection. Pathological mapping of the lungs of non-treated (NT) (**A**), deferred-treatment (DT) (**B**) and immediate-treatment (IT) (**C**) groups. Photomicrographs of the pathological Giemsa staining of the lung sections (100X). Mean histological severity score (HSS) was 2.64±0.4 (mean ± SD) for the NT group, 1.27±0.6 for the IT group (p = 0.005 *vs.* NT) and 2.32±0.4 for the DT group (p = NS *vs.* NT).

Notably, consistent with the increased survival rate observed in the IT group, we also observed that compared with the NT group, the damage to the lungs of the animals in the IT group was less significant (**Fig. 3**), as revealed by a significantly lower HSS (HSS IT group vs. NT group: 1.27±0.6 *vs*. 2.64±0.4; *p* = 0.005). In the DT group, the mean HSS was not significantly different from that in the NT group.

**Figure 4:**
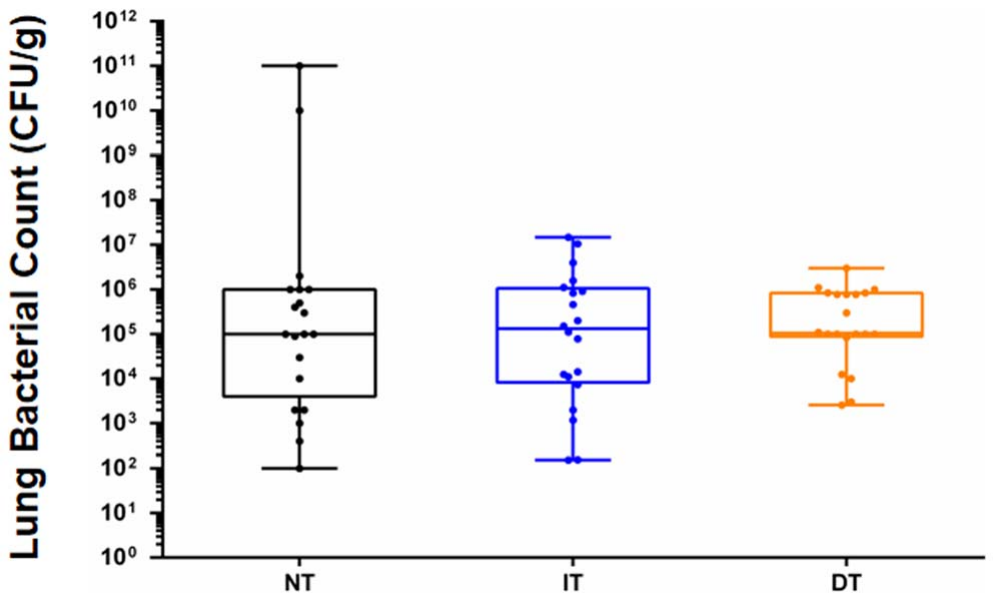
Lung bacterial count after infection. The lung bacterial count was adjusted according to the lung weight. The box plots limits represent the 25^th^ and 75^th^ percentiles, and the bars represent the 5^th^ and 95^th^ percentiles. The median is represented as a horizontal line. NT =  non-treated group; IT =  immediate treatment group; DT =  deferred treatment group.

Moreover, we also investigated the potential effects of lactonase treatment on the lung bacterial count, associated with the increased survival observed in the IT group. We observed that the lung bacterial count did not significantly differ between the 3 groups (CFU/g of lung in median [inter-quartile range]: 3.3×10^5^ [5.6×10^3^−1.3×10^6^] in the NT group; 1.3×10^5^ [9.2×10^3^−10^6^] in the IT group; and 10^5^ [8.4×10^4^−7.8×10^5^] in the DT group) (**Fig. 4**).

Finally, there was no difference concerning the number of animals with positive blood or spleen culture at the time of death between the 3 groups (n/tot (%): 8/20 (40%) in the NT group; 7/20 (35%) in the IT group; 10/20 (50%) in the DT group; p = NS).

## Discussion and Conclusions

In the present study, we used both *in vitro* and *in vivo* experiments to show that the lactonase *Sso*Pox-I was able to decrease the activity of *lasB* virulence gene, the synthesis of pyocianin, the proteolytic activity and the biofilm formation of the *P. aeruginosa* PAO1 strain. In addition, the treatment with *Sso*Pox-I was associated with an important improve in survival in a rat model of highly lethal acute pneumonia.

Originally, this work focuses on the lactonase *SsoPox*-I QQ activity with a design trying to approach clinical settings by giving the enzyme as a therapeutic drug. Similarly, QS inhibitor molecules such as furanones have been shown to increase the bacterial clearance in animal models of *P. aeruginosa* pneumonia but data on reduction of mortality are lacking [16] [19], [20]. In one study in mice using indirect demonstration of QQ effects of lactonases in animal models of pneumonia [31], Migiyama *et al*. showed a decrease in mortality and lung damage when animals were infected with a lactonase-producing *P. aeruginosa* strain. The main difference between Migiyama's study and ours is that we used a wild strain of *P. aeruginosa* for infections and we treated the animals with a synthetic lactonase given after infection as a therapeutic drug.

The catalytic efficiency and quenching activity of the QS lactonase *Sso*Pox-I against 3-oxo-C12 AHLs has been previously improved through protein engineering [32]. While the QS activity of the wild-type enzyme has been previously demonstrated *in vitro* [26], the efficiency of this enzyme *in vivo* has not been explored. *Sso*Pox-I inhibits the *lasB* gene activity, a classical virulence factor [41], at concentrations as low as 0.5 µg/ml. *Sso*Pox-I is also able to decrease the pyocianin synthesis as well as the proteolytic activity of a *P.aeruginosa* PAO1 strain. Moreover, the effect of *Sso*Pox-I on *P. aeruginosa* goes beyond the inhibition of virulence-associated genes. Indeed, *Sso*Pox-I also inhibited biofilm formation, albeit at much higher concentrations (170 µg/ml). This discrepancy in the active dose of the enzyme might reflect the distinct regulation of biofilm formation and maturation [12] [42]. Indeed, multiple quorum sensing-regulated genes are modulated without affecting lasRI, rhlRI or the production of N-acyl-L-homoserine lactones. In particular, the transcriptional regulator MvfR may contribute more to biofilm formation of *P. aeruginosa.* This could explain why in our experiments *Sso*Pox-I seemed to have a better efficacy on *lasB* gene down regulation than on decrease in biofilm formation [43], [44].

We further investigated the potential consequences of the observed *in vitro* effects. For this, we used an acute lethal model of *P. aeruginosa* pneumonia. Our model was consistent with previous ones showing a high and early lethality within the 24–48 first hours [45]–[47]. On histological analysis, the lungs of untreated animals exhibited important and confluent lesions of broncho-pneumonia arguing for the correlation between death and severity of pneumonia. In addition, nearly one half of the animals had bacteremia which probably contributed to the high mortality rate. In this model, *Sso*Pox-I significantly decreased the mortality rate from 75% in the non-treated group to 20% in the group treated immediately after infection. This observation is consistent with the dramatic reduction in the lung damage observed in the treated group. The results are also consistent with previous studies showing that infections with QS-deleted strains of *P. aeruginosa* were less severe in several infection models [48] [49].

*Sso*Pox-I did not significantly reduce lung lesions and rat mortality when administrated at 3 hours after the onset of infection. This ineffectiveness might be due to the use of a highly lethal model of infection (death in 48 hours). The preventive action of *Sso*Pox-I might be higher than its curative effect as previously observed for QSI [22]. QS is indeed under a positive retro-control regulation [50]: when the bacterial *quorum* is reached, the inhibition of QS is more difficult because of the self-stimulating properties of bacteria.

In our study, the innovative molecule *Sso*Pox-I was administered intra-tracheally within the 3 first hours of infection. This design was chosen to approach clinical preventive therapeutic methods used in patients at risk for *P. aeruginosa* infections, such as ICU patients. However, our model did not totally mimic clinical settings primarily because of the high amount of bacteria given in one inoculation.

No difference in the lung bacterial burden was observed between the control group and the 2 treated groups regardless the time of administration of the molecule. While surprising, these results are consistent with our *in vitro* findings on bacterial cultures in which lactonase adjunction to the media did not influence *P. aeruginosa* growth. These results agree with those of Migiyama *et al*. [31] who showed similar bacterial count with animals infected with wild-type *P. aeruginosa* and their lactonase-producing mutant strain. It remains however unknown whether the improved survival could be due to a less invasive activity of the microorganism towards the lung parenchyma and/or to change in the host-pathogen interaction regardless the bacterial load.

In summary, *Sso*Pox-I presents several properties that could increase the therapeutic arsenal, particularly in the field of nosocomial pneumonia. In contrast to antibiotics, *quorum*-quenching strategies do not impose drastic selection pressure on bacterial survival. Therefore, treatments with lactonases such as *Sso*Pox-I might not or only slightly, promote the emergence of resistance [7] [51]. Interestingly, due to their ability to reduce biofilm formation, lactonases could restore susceptibility to antibiotics in drug-resistant strains as previously observed with tobramycin in animal models [52]. Because of its mechanisms of action involving a modulation of the QS, azythromycin has been recently shown in a randomized controlled trial to reduce the incidence of ventilator-acquired pneumonia in pseudomonas colonized patients [53] showing the growing interest of QS inhibitors in clinics.

To conclude, our results open the way to further investigations assessing *Sso*Pox-I as a possible tool in antimicrobial strategy.

## Supporting Information

Figure S1 **SDS-PAGE of** ***Sso*** **Pox-I.** Twenty-five µg of *Sso*Pox-I (left band) were deposited next to a Molecular weight Marker (MwM, right panel) (Mulicolor broad range protein ladder, Euromedex).
(TIF)

Figure S2 **The growth of** ***P. aeruginosa*** **in the presence of** ***Sso*** **Pox-I.** A small decrease in *P. aeruginosa* growth was observed at the highest concentration of *Sso*Pox-I; however, this effect was not significant (Student's t test p = 0.67). The chart shows percentage of controls (no *Sso*Pox-I) and represents the data obtained from four independent experiments, each performed with three technical replicates. The error bars represent 95% confidence intervals.
(TIF)

Checklist S1 **Arrive guidelines.**
(PDF)
