# Supplementary figures and images for: Inhaled Lactonase Reduces Pseudomonas aeruginosa Quorum Sensing and Mortality in Rat Pneumonia
Source: PLoS One. 2014 Oct 28;9(10):e107125. doi: 10.1371/journal.pone.0107125 (PMC4211673; doi:10.1371/journal.pone.0107125)

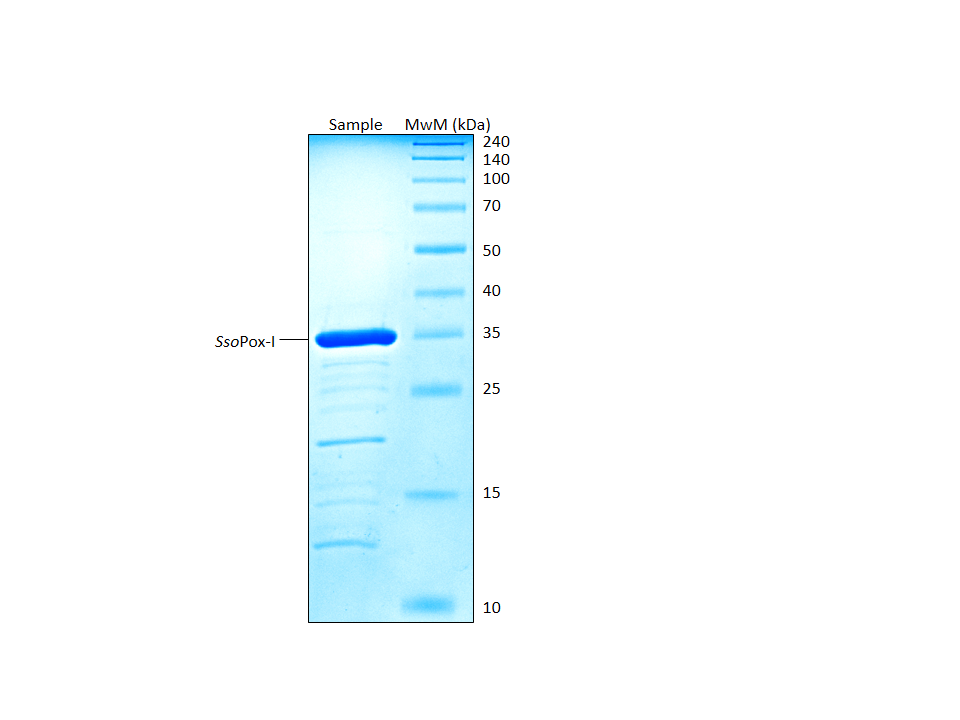

Supplement: Figure S1 — SDS-PAGE of Sso Pox-I. Twenty-five µg of SsoPox-I (left band) were deposited next to a Molecular weight Marker (MwM, right panel) (Mulicolor broad range protein ladder, Euromedex). (TIF) [file pone.0107125.s001.tif]

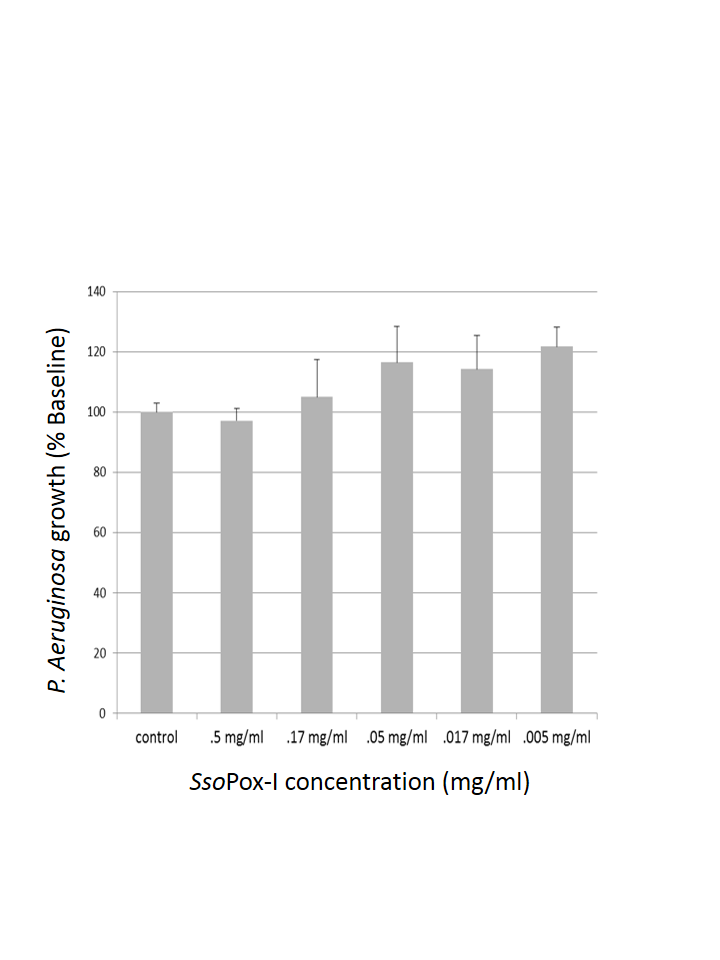

Supplement: Figure S2 — The growth of P. aeruginosa in the presence of Sso Pox-I. A small decrease in P. aeruginosa growth was observed at the highest concentration of SsoPox-I; however, this effect was not significant (Student's t test p = 0.67). The chart shows percentage of controls (no SsoPox-I) and represents the data obtained from four independent experiments, each performed with three technical replicates. The error bars represent 95% confidence intervals. (TIF) [file pone.0107125.s002.tif]
